# Smart Phone, Smart Science: How the Use of Smartphones Can Revolutionize Research in Cognitive Science

**DOI:** 10.1371/journal.pone.0024974

**Published:** 2011-09-28

**Authors:** Stephane Dufau, Jon Andoni Duñabeitia, Carmen Moret-Tatay, Aileen McGonigal, David Peeters, F.-Xavier Alario, David A. Balota, Marc Brysbaert, Manuel Carreiras, Ludovic Ferrand, Maria Ktori, Manuel Perea, Kathy Rastle, Olivier Sasburg, Melvin J. Yap, Johannes C. Ziegler, Jonathan Grainger

**Affiliations:** 1 CNRS, UMR 6146, Marseille, France; 2 Aix-Marseille Université, UMR 6146, Marseille, France; 3 Basque Center on Cognition, Brain and Language, Donostia, Spain; 4 Universidad Católica de Valencia, Valencia, Spain; 5 INSERM, U751, Marseille, France; 6 International Max Planck Research School for Language Sciences, Nijmegen, The Netherlands; 7 Washington University in St. Louis, St. Louis, Missouri, United States of America; 8 Universiteit Gent, Gent, Belgium; 9 IKERBASQUE, Basque Foundation for Science, Bilbao, Spain; 10 CNRS and University Blaise Pascal, Clermont-Ferrand, France; 11 Universitat de València, València, Spain; 12 Royal Holloway, University of London, Egham, United Kingdom; 13 National University of Singapore, Singapore, Singapore; Chiba University Center for Forensic Mental Health, Japan

## Abstract

Investigating human cognitive faculties such as language, attention, and memory most often relies on testing small and homogeneous groups of volunteers coming to research facilities where they are asked to participate in behavioral experiments. We show that this limitation and sampling bias can be overcome by using smartphone technology to collect data in cognitive science experiments from thousands of subjects from all over the world. This mass coordinated use of smartphones creates a novel and powerful scientific “instrument” that yields the data necessary to test universal theories of cognition. This increase in power represents a potential revolution in cognitive science.

## Introduction

The number of smartphone users worldwide is expected to exceed one billion by 2013. Undoubtedly, smartphones have become a part of our daily lives. By taking advantage of such massive use, we show how these miniature pocket computers can revolutionize research in cognitive science.

Investigating human cognitive faculties such as language, attention, and memory most often involve small groups of volunteers coming to a research facility where they are asked to participate in behavioral experiments under a controlled environment. Previous attempts to make conventional laboratory experiments accessible to a wider audience, with use of internet-based technologies [1], [2], have not provided the necessary temporal precision for understanding the millisecond changes in cognitive processes. Internet-based research is thus unsuitable for many behavioral experiments that require such precision [3], [4]. Indeed, precision in the measurement of stimulus duration (e.g. image display) and behavioral responses (e.g. pushing a button) has been of utmost importance ever since the seminal research in experimental psychology at the end of the 19th century [5], [6].

In contrast, smartphone technology (as in iPhone/iPad) offers high temporal and spatial resolution with built-in millisecond timing of stimuli display and touchscreen responses. Individual iPhones are tools that are portable, easy to use, multimedia-enabled and identical in every country and for each user, with ready Internet transfer of collected data. These properties render it an instrument ideally adapted to studying cognitive functions. However, the real revolution arises from the mass co-ordinated use of smartphones on a worldwide level, harnessing the power of precise technical specifications to create a novel multi-dimensional scientific “instrument” capable of experimentation on a previously unthought-of scale. Indeed, as for other scientific disciplines (e.g., Very Long Baseline Interferometry in Astronomy), increasing the power of the scientific tool makes it possible to detect novel phenomena and expand previous analyses. Likewise, use of smartphones allows us to dramatically increase the amount of data collected without sacrificing precision, and thus has the potential to uncover laws of mind that have previously been hidden in the noise of small-scale experiments.

## Results and Discussion

**Figure 1:**
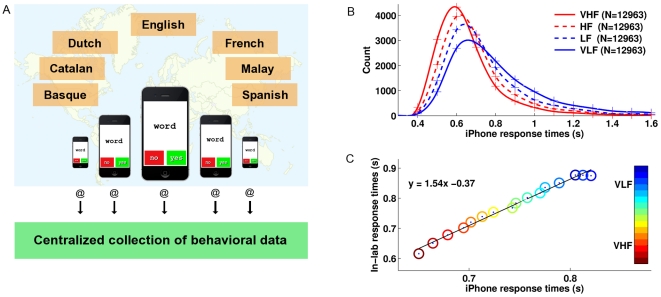
Scientific investigation of human behavior using iPhone. (A) Illustration of the large scale scientific instrument for the study of human behavior. (B) Histograms of correct response times on English words as a function of word frequency (very high frequency words such as “WITH”, VHF; high frequency, HF; low frequency, LF; very low frequency such as “ORANGEADE”, VLF). Histograms were computed using 100 ms bins (“+” signs linked by a spline interpolation function). (C) Scatterplot of mean correct response times on words binned by word frequency in groups of 2500.

We report an innovative method of using iPhone/iPad devices to conduct a classic cognitive psychology task in the field of psycholinguistics, the lexical decision task [7], [8] (see Figure 1A). This task has historically provided considerable insight into the cognitive processes involved in skilled reading as well as reading impairments such as dyslexia, through measuring millisecond-level response time and accuracy in deciding if a letter string is a word (“TABLE”) or not (i.e. a nonword, such as “TIBLE”). Participants within the general public download this newly developed application free of charge from the App Store, perform one or more sessions of 50 to 140 randomly selected stimuli and may voluntarily provide additional data such as age, gender, handedness and native language. Participants are finally invited to submit responses by e-mail. The data collected so far show that response time distributions are strikingly similar to those obtained in laboratory conditions and predicted by mathematical models of decision processes [9]–[11] (i.e., right-skewed normal distribution; Figure 1B). The word frequency effect is reflected in both the decreased skew of the distribution and the shift of the median as word frequency increases. Compared to an in-laboratory lexical decision experiment in English [12], iPhone response times show a linear relationship (Figure 1C).

It could be argued, given that many smartphone users are younger generation, that a sampling bias may thus be introduced in the current methodology. However, we would argue that, on the contrary, the use of smartphones in the present study has allowed us to reach a more diverse population than is typically represented in most existing psycholinguistic experiments, the majority of which rely on undergraduate students from research intensive universities. Moreover, the fact that participants can report additional data such as age or gender allows for statistical analysis of either a population sub-group or the whole population by including such variables in a general regression model.

This project was launched as a multi-language international collaboration in December 2010, with data so far from 4157 participants (four months) for the seven language-specific versions of the application. In comparison, it took more than three years to collect a similar amount of data in the largest currently available database on word-reading [12]. While the present study involves seven languages based on the Roman alphabet (English, Basque, Catalan, Dutch, French, Malay, Spanish), this could readily be expanded to include other languages including those using different alphabets and scripts (smartphones natively support Chinese, Greek, and Japanese, for example). A specific and unique interest of the present project is the ability to perform large-scale cross-linguistic comparisons, hitherto impossible in this domain. The results obtained provide normative data on skilled reading across countries that amongst other uses could assist in the diagnosis and remediation of reading impairments.

The use of smartphones for scientific experimentation heralds a new era in behavioral sciences. The approach has wide multidisciplinary applications in areas as diverse as economics, social and affective neuroscience, linguistics, and experimental philosophy. Finally, it becomes possible to reliably collect culturally diverse data on a vast scale, permitting direct tests of the universality of cognitive theories.

## Materials and Methods

### Stimuli databases

Stimuli were the English words and nonwords of the English Lexicon Project database [12].

### Procedure and task

Participants were asked to select one of the three proposed session durations (50, 100 or 140 stimuli corresponding roughly to 2-, 4- or 6-minute sessions). Sessions were composed of an equal mix of words and nonwords randomly drawn from the database. Stimuli were displayed in black (Courier New Bold, 24 points) in the middle of a white background screen. The screen also displayed two response buttons (YES and NO) in its lower right and left corners. A trial consisted of a fixation cross displayed for 0.3 seconds followed by a stimulus displayed until a response was given. Participants were asked to press the YES button if the stimulus was a word, or NO otherwise. In the absence of a response after a 1.7 second time-out, the sentence “Please, answer more quickly” was displayed. At the end of a session, participants were asked to indicate their gender, age category, handedness and native language. A results page was then displayed providing the mean accuracy and response time of the session. Participants could finally send their recorded data by e-mail.

### Response times and accuracy data

Response times and accuracy of items were extracted from 4157 emails received during the four-month period following launch of the application. Sessions with mean accuracy below the global mean accuracy minus two standard deviations were discarded from further analysis. Correct word trials were selected. Response times calculated from stimulus display onset that were above 0.3 and below 1.7 seconds were kept in the analysis. Overall, 8.70% of the English word trials were rejected.

The investigation was conducted using seven language-specific versions of the application for iPhone/iPad/iPod. Application was organized as a word game. Information about the nature of the word game was given in the iTunes App Store. On a voluntary basis, participants downloaded the application free of charge, performed one or more game sessions, and were invited to submit their records by email. Before performing the word test, the instructions and consent page was displayed on screen. Participants were required to click “yes” to indicate their agreement to the study before using the application. Consent was thus obtained electronically from each participant, due to the nature of the study. This research (ERC#230313), including the method of consent, was approved by the internal review board of the Université de Provence.
